# Prevention and Control of COVID-19 Infection in a Chinese Mental Health Center

**DOI:** 10.3389/fmed.2020.00356

**Published:** 2020-07-03

**Authors:** Mi Yang, Hongming Wang, Zhi Li, Qiang Zhang, Xin Liu, Manxi He, Shan Gao

**Affiliations:** ^1^Chengdu Mental Health Center, Chengdu, China; ^2^The Fourth People's Hospital of Chengdu, Chengdu, China; ^3^The Clinical Hospital of Chengdu Brain Science Institute, MOE Key Laboratory for NeuroInformation, University of Electronic Science and Technology of China, Chengdu, China; ^4^College of Electronics and Information Engineering, Sichuan University, Chengdu, China; ^5^School of Foreign Languages, University of Electronic Science and Technology of China, Chengdu, China

**Keywords:** the 2019 novel coronavirus disease (COVID-19), outbreak, mental health center, nosocomial infection, prevention and control practice

## Abstract

Faced with the rapid spread of the novel coronavirus disease (COVID-19), a global public health threat, psychiatric hospitals are under huge pressure to prevent and control nosocomial infection. The current research analyzed the COVID-19 infection control practices in a regional mental health center in China and addressed how this type of medical institutions could enhance their ability to prevent and control hospital transmission of major respiratory diseases and general management of nosocomial infection risks. Firstly, hospital-related risks of COVID-19 were analyzed, and targeted prevention and control measures were then established. Pre- and post-intervention theoretical knowledge of nosocomial infection control, hand hygiene compliance and accuracy, use of personal protective equipment, and disinfection and sterilization effectiveness were evaluated and compared. All the indexes displayed significant improvements following the implementation of the prevention and control measures. Up to the submission of this paper, the mental health center had obtained no suspected or confirmed case of COVID-19 infection due to hospital transmission. The findings provide empirical evidence for the effectiveness of the COVID-19 preventive strategies and have important implications for integrated and characterized infection control in mental health centers during a major epidemic. The establishment of the transitional isolation ward and air fumigation using traditional Chinese medicine for patients and staff are preventive measures worthy of further discussion and dissemination.

## Introduction

The 2019 novel coronavirus disease (COVID-19) epidemic has spread rapidly worldwide since it was first reported in Wuhan, China, on the 31st of December, 2019 (1). It consequently became a global public health threat and was characterized as a pandemic by the World Health Organization (WHO) on the 11th of March, 2020 (2). COVID-19 is highly infectious, and it has an estimated mean reproductive number (*R*_0_) of around 3.28 according to an early review (3). Hospital transmission of the virus can be a major contribution to the spread of the disease (4). Therefore, healthcare settings have been under huge pressure to prevent and control nosocomial infection (5). Such pressure is even heavier in psychiatric hospitals where patients are particularly vulnerable: (a) wards are not zoned for infection control and often managed in a closed manner, with restricted space for activities and poor quality of indoor air; (b) antipsychotics, with functions of sedation and muscle relaxation, inhibit the movement of respiratory cilia and thus weaken the ability of the respiratory tract to eliminate pathogenic bacteria; and (c) the forced supine position of constrained patients may lead to enhanced vulnerability to respiratory infection. In addition, psychiatric patients lack the sense of self-protection or the desire for treatment, which increases the difficulty in epidemic prevention and the likeliness of delayed treatment. On the other hand, staff (particularly non-medical staff) in psychiatric hospitals do not have sufficient awareness and knowledge of coping with infectious diseases. Nosocomial spread of COVID-19 in Wuhan Mental Health Center, where 50 patients and 30 medical staff were diagnosed with COVID-19 as of the 8th of February (6), indeed suggests it is very urgent to initiate a prevention and control system for psychiatric hospitals in the face of major respiratory infectious diseases. During the COVID-19 epidemic, therefore, the Chengdu Mental Health Center (CMHC, also named the Fourth People's Hospital of Chengdu, Chengdu, China) has taken a number of measures in its practice to prevent and control nosocomial infection. The present study aims to analyze the COVID-19 infection control practices in the mental health center and evaluated their effectiveness with empirical evidence to address how this type of medical institutions could enhance their ability to prevent and control hospital transmission of major respiratory diseases and general management of nosocomial infection risks.

## Materials and Methods

### Assessment of Nosocomial Infection Risks and Development of Countermeasures

**Figure 1:**
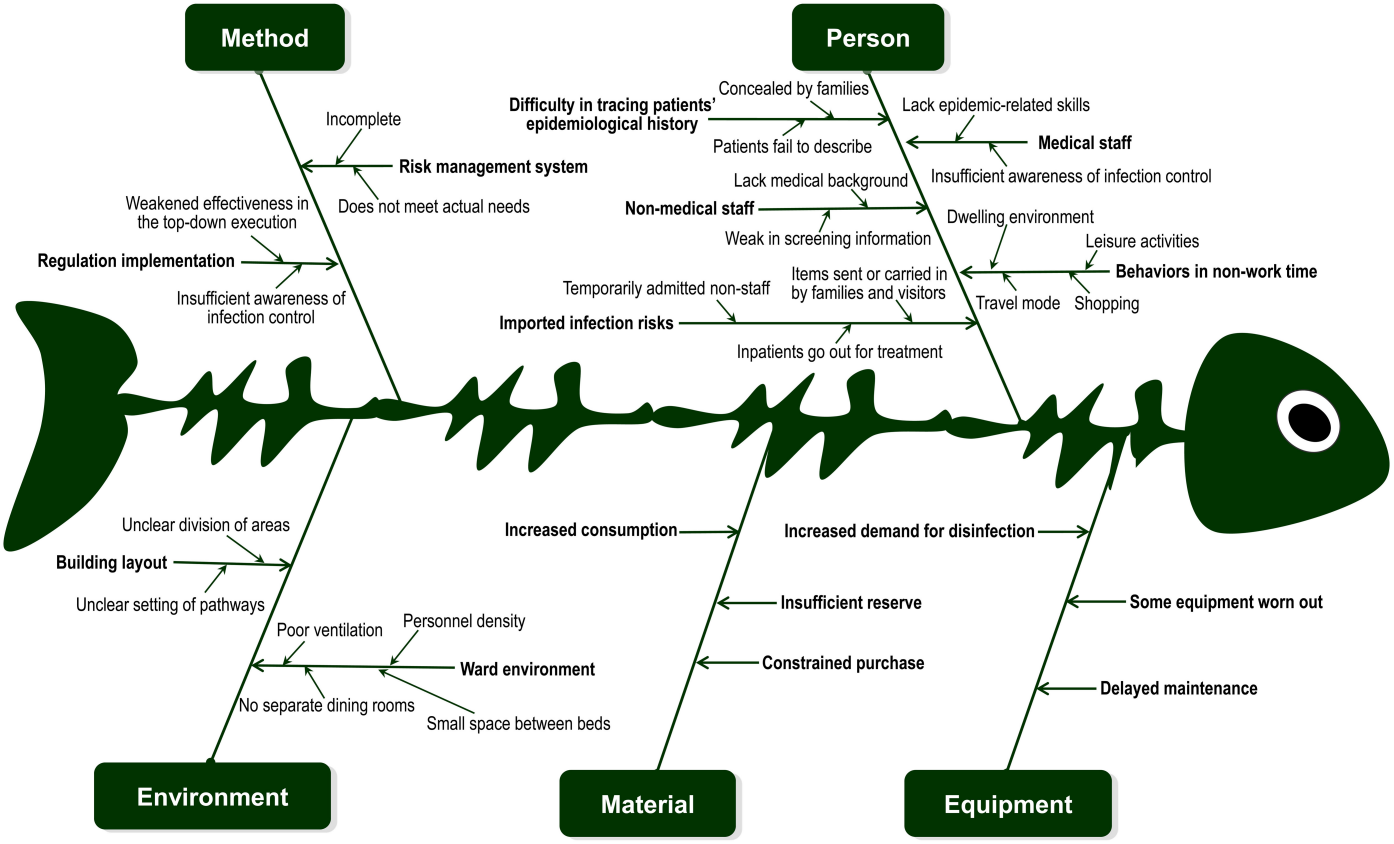
Risks of nosocomial infection in Chengdu Mental Health Centre during the COVID-19 epidemic. Risks of nosocomial infection were analyzed in relation to five aspects (person, equipment, material, method, and the environment), and corresponding prevention and control strategies were established.

Risks of hospital infection of COVID-19 in CMHC were assessed, and corresponding measures have subsequently been initiated to reduce the risks following the Technical Guidelines for the Novel Coronavirus Infection Prevention and Control in Medical Institutions (First Edition) (7). We took “risk control of nosocomial infection during the COVID-19 epidemic” as a target product and employed five quality contributor categories from the perspective of total quality management (8), namely, person, equipment, material, method, and the environment (Figure 1), to analyze risks and establish corresponding coping strategies.

(a) The risk control system was neither sound nor well-implemented.

The epidemical prevention and control system has pervasively been under development in psychiatric hospitals, including the CMHC. Therefore, coping with such major respiratory infectious diseases as COVID-19, the CMHC has been short of detailed plans, procedures, and guidelines. Also, practical needs were not fully taken into consideration.

Measures: Instructions issued by higher authorities were followed (7), a documented infection control system, initiated by the CMHC, suitable, and operable for the center by investigating front-line conditions, was implemented, and coping strategies were summarized and discussed repeatedly. This strategic system was constantly modified and updated with the development of the epidemic.

On the other hand, regulations and measures are not well-implemented. The effectiveness of the infection control instructions determined by higher authorities was weakened when the measures were implemented at lower levels, particularly in the clinical first-line.

Measures: Each division selected an anonymous infection control inspector. The Department of Infection Control in the CMHC gave the inspectors intensive training and appointed them to disseminating and inculcating the knowledge about COVID-19 and nosocomial infection as well as supervising infection control implementation in their division.

(b) Personnel management was the most unstable and risky aspect in preventing and controlling hospital infection.

Firstly, it was hard to trace the precise epidemiological history of newly admitted or returned patients due to their psychiatric symptoms. On the other hand, patients' families were in sore need of having patients treated in hospital and thus might conceal part of patients' epidemiological history.

Measures: Patients were admitted to the regular ward after 14 days of quarantine and observation in the transitional zone.

Secondly, medical staff in the psychiatric specialty were not fully qualified to diagnose and treat COVID-19. “Instant” training could not instantly improve their competence in infection, internal medicine, and epidemiology. Additionally, they did not have sufficient awareness of infection control, which added difficulty to effective implementation of infection control measures.

Measures: Medical specialists and nosocomial infection commissioners were assigned to give repeated training to staff groupings through the use of video, desktop deduction, model-based operation, and onsite demonstration. Then, the training effectiveness was assessed by online tests of different levels.

Thirdly, non-medical personnel lacked skills in infection prevention and control.

Without a medical background, administrative and logistic personnel, particularly low-educated nursing attendants, cleaning workers, security, and canteen staff, were weak in discerning epidemic information and thus had two extreme attitudes, namely, “excessive tension” and “blind optimism,” leading to a negative influence on implementation of prevention and control measures.

Measures: Key information in classified training was highlighted, popularized, and visualized during the onsite explanation and demonstration. Guidance and supervision were then repetitively given. Besides this, psychological counseling was provided.

Fourthly, it was difficult to manage behaviors of staff outside of working hours.

Staff might have a variety of activities out of the hospital such as taking public transportation, renting houses with others, having parties, and going shopping when they failed to self-monitor and strictly implement the prevention and control measures, leading to increased risks of imported infections in the hospital.

Measures: Staff was suggested to travel by private cars as much as possible. If it was not possible, they were suggested to walk or bike within 5 km or take a taxi or share a taxi within groups of staff. Daily registration was initiated, monitoring temperature and location of staff. Commitments of responsibility were signed to reinforce self-regulation awareness.

Fifthly, there were imported infection risks to inpatients.

The virus might be brought in to hospitalized patients when non-staff members, such as patient escorts, oxygen delivery men, consultation doctors, maintenance servicemen, and information technicians, entered the ward. On the other hand, inpatients might need to go out for occupational and recreational treatments and other supplementary treatments (e.g., modified electroconvulsive treatment and ultrasonography). Families and visitors might bring patients personal items by having them delivered or personally carrying them in.

Measures: Personnel mobility in the ward was reduced by advocating bedside inspection, telephone- and internet-based consultation, and video visitation. If examinations and treatments had to be done out of the hospital, masks were required, and cohorts of patients were allowed to go out at different times following a pre-specified route. The inspection department took care of disinfection afterwards. Articles sent by couriers or visitors were effectively disinfected before brought into the ward. Quick hand disinfectant was provided at the entrance and exit of the hospital as well as heavily congested places including the outpatient hall, elevators, etc.

(c) The reserves of protective materials and disinfectant equipment were limited.

The supplies of protective materials, such as surgical masks, medical protective masks, medical protective clothing, and isolation clothing, were insufficient compared to the demands of the COVID-19 epidemic. An increasing number of air disinfectant equipment and terminal sterilizers were needed.

Measures: Statistics of the first-level and second-level reserves of all prevention and control materials and storage locations of relevant equipment were updated on a daily basis. The approval for access to the materials and equipment were restricted so as to ensure a sufficient supply of materials and equipment in the emergency disposal of the clinical first-line.

(d) There were environmental risks of nosocomial infection.

The building layout and ward environment did not meet the requirements of hospital infection prevention and control. The potential contamination area and contamination area in the ward were not strictly separated, leading to higher risks of cross infection. It is difficult to set up emergency quarantine rooms and buffer zones in the regular ward. It is also difficult to achieve adequate ventilation and implement quarantine measures since the ward is densely occupied and the space between beds is small.

Measures: the CMHC quickly modified the ward layout and set up “three areas” and “three channels” (channels for regular patients, feverish patients, and medical staff). Different areas were chosen in the hospital, respectively, for suspected and confirmed but mild cases of COVID-19, for transitional quarantine, and for regular fever observation. New patients who did not have a fever or those who returned from a pre-arranged leave of absence were kept in the transitional quarantine and transferred to the regular ward after 14 days of observation. Patients with a fever that was not related to COVID-19 were kept individually in a quarantine room and transferred to the regular ward when they maintained normal in body temperature for at least 3 days and were evaluated as admissible.

There were also deficiencies in environmental management. Despite the enclosed management of inpatients, the ward was still exposed to infection risks since it was densely occupied and lacked in sufficient ventilation. There were no separate dining rooms. Patients of psychoses did not cooperate in wearing masks.

Measures: Windows were open for ventilation and air was disinfected as required. Moreover, air fumigation with traditional Chinese medicine (9) was used to prevent air transmission the novel coronavirus. Single isolation rooms were set up in the regular ward in case of emergency use for feverish patients before the patients were decided to transfer to the fever ward.

### Evaluation of the Effects of Infection Prevention and Control Measures

The present study examined the effects of the system of measures during their implementation between the 17th of January and the 10th of March, 2020. Before and after the intervention, 205 doctors, 475 nurses, 138 nursing attendants, 51 cleaners, and 35 security guards were evaluated in terms of theoretical knowledge of nosocomial infection control, hand hygiene implementation, use of personal protective equipment, and disinfection and sterilization effectiveness. All evaluation procedures were administered by an infection control team of 26 members (four were full-time and had a nursing, epidemiology, and statistics background). The research was approved by the Ethics Committee of the Fourth People's Hospital of Chengdu (approval number 2020-19).

- Theoretical knowledge of nosocomial infection was tested via authorized software.
- Based on the Chinese version of the observation sheet of hand hygiene compliance issued by WHO (10), hand hygiene compliance was examined in staff without examinees' knowledge to eliminate the “Hawthorne effect” (11). The compliance rate was obtained with actual times of hand washing divided by hand hygiene opportunities and multiplied by 100%. The accuracy of hand hygiene in staff was also observed and recorded by infection control inspectors (11).
- Based on the Technique Standard for Isolation in Hospital (WS/T 311-2009) (12), the accuracy of personal protection implementation was assessed.
- The Regulation of Disinfection Technique in Healthcare Settings (WS/T 367-2012) (13) was used as the standard to evaluate the quality of sterilization.

All data were analyzed using SPSS 26. Count data were described by frequency percentage. χ^2^*-*tests were used to compare all the rates before and after the intervention, and *P* < 0.05 was considered significant.

## Results

### Qualification Rate of Theoretical Knowledge of Nosocomial Infection

Prior to the intervention, 468 of 732 staff passed the examination of theoretical knowledge of nosocomial infection, with a passing rate of 63.93%. Following the intervention measures, the total qualification rate rose up to 87.28% (χ^2^ = 16.52, *P* < 0.001, 95% CI 1.18–1.59%), and the qualification in each type of personnel (all *P*s < 0.05; Table 1) was also significantly improved.

**Table 1:** Qualification rate of theoretical knowledge of nosocomial infection before and after intervention.

**Position**	**Before intervention**			**After intervention**			**χ^**2**^**	***P***	**CI**	
	**Participants (*n*)**	**Qualified (*n*)**	**Qualified (%)**	**Participants (*n*)**	**Qualified (*n*)**	**Qualified (%)**			**Lower**	**Upper**
Doctors	169	105	62.13	205	183	89.27	5.11	0.024	1.05	1.97
Nurses	413	305	73.85	475	436	91.79	4.69	0.030	1.02	1.51
Nursing attendants	86	35	40.70	138	97	70.29	5.22	0.022	1.08	2.77
Cleaners	37	13	35.14	51	42	82.35	5.04	0.025	1.11	4.97
Security guards	27	10	37.04	35	31	88.57	3.94	0.017	1.00	5.72
Total	732	468	63.93	904	789	87.28	16.52	<0.001	1.18	1.59

### Compliance Rate and Accuracy of Hand Hygiene

A total of 3,246 hand hygiene opportunities were observed before and after the intervention. In practice, 817 and 1,440 hand cleansing cases occurred before and after the intervention, respectively. The pre-intervention hand hygiene compliance was 56.97%. Following the intervention measures, the compliance rate rose up to 79.47%, indicating a significant increase of overall hand hygiene compliance (χ^2^ = 35.06, *P* < 0.001, 95% CI 1.25–1.56%). Indeed, a significant improvement of hand hygiene compliance was found in each type of personnel in the investigation including doctors, nurses, nursing attendants, cleaners, and security guards (all *P*s < 0.05; Table 2). In addition, prior to the intervention, 817 cases of hand hygiene occurred, and 459 of them were performed accurately, producing an accuracy of 56.18%. After the intervention, 1,166 of 1,440 (80.97%) hand hygiene practices were identified as accurate, indicating a significant improvement via the intervention (χ^2^ = 27.08, *P* < 0.001, 95% CI 1.26–1.65%).

**Table 2:** Hand hygiene compliance before and after intervention.

**Position**	**Before intervention**			**After intervention**			**χ^**2**^**	***P***	**CI**	
	**Opportunities (*n*)**	**Practices (*n*)**	**Compliance (%)**	**Opportunities (*n*)**	**Practices (*n*)**	**Compliance (%)**			**Lower**	**Upper**
Doctors	408	251	61.52	534	417	78.09	5.32	0.021	1.04	1.56
Nurses	611	450	73.65	763	675	88.47	5.06	0.025	1.02	1.41
Nursing attendants	205	57	27.80	289	193	66.78	25.44	<0.001	1.70	3.94
Cleaners	121	34	28.10	125	83	66.40	13.13	<0.001	1.48	3.78
Security guards	89	25	28.09	101	72	71.29	11.91	0.001	1.48	4.34
Total	1,434	817	56.97	1812	1,440	79.47	35.06	<0.001	1.25	1.56

### Accuracy of Personal Protection Implementation

A total of 982 cases of putting on and taking off protective clothing and articles were observed, including 391 and 591 cases, respectively, before and after the intervention. Prior to the intervention, 131 of the observed cases were performed accurately, with accuracies of 28.95 and 37.81% for putting on and taking off, respectively. Via the intervention measures, 504 of the observed cases were performed accurately, leading to strikingly increased accuracies for putting on (82.55%; χ^2^ = 36.33, *P* < 0.001, 95% CI 2.01–4.04%) and taking off (87.66%; χ^2^ = 29.09, *P* < 0.001, 95% CI 1.7–3.16%). In fact, the accuracies were improved greatly for putting on and taking off all kinds of protective equipment, including medical protective masks, medical isolation, and protective clothing (all *P*s < 0.05; Table 3).

**Table 3:** Accuracy of personal protection implementation before and after intervention.

**Protective equipment**		**Before intervention**			**After intervention**			**χ^**2**^**	***P***	**CI**	
		**Examined cases (*n*)**	**Accurate cases (*n*)**	**Accuracy (%)**	**Examined cases (*n*)**	**Accurate cases (*n*)**	**Accuracy (%)**			**Lower**	**Upper**
Medical protective mask	Put on	55	21	38.18	89	75	84.27	7.09	0.008	1.22	3.98
	Take off	67	34	50.75	101	87	86.14	4.30	0.038	1.03	2.81
Medical isolation clothing	Put on	47	17	36.17	72	57	79.17	5.62	0.018	1.14	4.21
	Take off	62	29	46.77	80	69	86.25	4.88	0.027	1.07	3.18
Medical protective clothing	Put on	88	17	19.32	114	95	83.33	26.08	<0.001	2.40	7.75
	Take off	72	13	18.06	135	121	89.63	27.34	<0.001	2.62	9.41
Total	Put on	190	55	28.95	275	227	82.55	36.33	<0.001	2.01	4.04
	Take off	201	76	37.81	316	277	87.66	29.09	<0.001	1.70	3.16

### Qualification Rate of Disinfection by Cleaning Workers

Before the intervention, the process and effect of disinfection were evaluated in 258 cleaning cases and 91 of them were considered qualified, with a qualification rate of 35.27%. The intervention measures led to a sharp increase in either the overall qualified rate (83.18%; χ^2^ = 35.1, *P* < 0.001, 95% CI 1.77–3.14%) or the qualified rates in all disinfection procedures, including preparation of disinfectant, ground disinfection, surface disinfection of frequently touched objects, tableware disinfection, and sanitary ware disinfection (all *P*s < 0.05; Table 4).

**Table 4:** Qualification rate of disinfection by cleaning workers before and after intervention.

**Disinfection procedures**	**Before intervention**			**After intervention**			**χ^**2**^**	***P***	**CI**	
	**Examined cases (*n*)**	**Accurate cases (*n*)**	**Accuracy (%)**	**Examined cases (*n*)**	**Accurate cases (*n*)**	**Accuracy (%)**			**Lower**	**Upper**
Disinfectant preparation	55	25	45.45	63	59	93.65	5.82	0.016	1.14	3.72
Ground disinfection	48	18	37.50	72	56	77.78	5.01	0.025	1.09	3.95
Object surface disinfection	64	22	34.38	81	63	77.78	7.63	0.006	1.26	4.06
Tableware disinfection	44	13	29.55	57	51	89.47	9.37	0.002	1.47	6.25
Sanitary ware disinfection	47	13	27.66	60	48	80.00	8.65	0.003	1.41	5.95
Total	258	91	35.27	333	277	83.18	35.10	<0.001	1.77	3.14

## Discussion

The present study addressed the risks of nosocomial infection of COVID-19 and corresponding measures in psychiatric hospitals, based on the experience of CMHC, a representative institution of mental health service in southwest China. While suggestions were given on certain aspects of hospital infection control for COVID-19 (6, 14–17), we recruited the concept of total quality management (8) to obtain systematic monitoring of infection risks from the perspectives of person, equipment, material, method, and environment, which led to integrated and targeted measures for epidemic prevention and control. Moreover, we evaluated the intervention effect. Significant improvements in theoretical knowledge of nosocomial infection control, hand hygiene compliance and accuracy, proper use of personal protective equipment, and disinfection and sterilization effectiveness were found after the intervention, indicating considerable achievements by establishing and implementing the integrated system of infection control strategies. Indeed, up to the submission of this paper, in CMHC there was no suspected or confirmed case of COVID-19 due to nosocomial infection. These results have important implications for characterized infection control in mental health centers.

In a mental health center like the CMHC, both medical and non-medical staff lack sufficient awareness and professional knowledge of COVID-19 infection prevention and control, which requires reinforced dissemination and training of the knowledge and skills. The increased qualification rate of theoretical knowledge examination here indicates good performance of repetitive training, which also facilitates the implementation of hand hygiene, use of personal protective equipment, disinfection, and sterilization. Hand hygiene is considered to be the most effective strategy to combat hospital-related infection and a considerable number of studies reported a reduction in infection rates after improved compliance with hand hygiene (18). Therefore, the improvement in the compliance and accuracy of hand hygiene suggests the effectiveness of infection prevention knowledge dissemination and measure implementation. Previous evidence has revealed that healthcare workers, even involved in the management of infectious disease, are not necessarily skilled in using personal respiratory protective equipment (19). This was also the case for the staff in the CMHC. After the intervention, however, the accuracy for using the equipment was greatly improved. On the other hand, the enclosed management of wards and vulnerability of patients make disinfection and sterilization highly demanded in mental health centers. Many studies have shown ineffectiveness of disinfection and sterilization in medical hospital material management (20). Indeed, the qualification rate of disinfection and sterilization in CMHC was very low before the infection control measures were implemented. Nevertheless, it was largely increased following the intervention. Taken together, the findings here provide empirical evidence for the effectiveness of the preventive strategies for nosocomial transmission during the COVID-19 pandemic, which has rarely been assessed despite the early proposal of infection control measures for both psychiatric hospitals (6) and healthcare institutions of other types (14–17).

Since the COVID-19 outbreak, CMHC has initiated an integrated strategic system of infection prevention and control based on the analysis of the epidemic and the experience of specialists in psychiatry, respiratory medicine, hospital infection, and other related fields. It is also an organizational system involving different departments to implement emergency protection regulations, infection control measures, risk assessment and analysis, multi-disciplinary cooperation, and targeted education and evaluation. As a designated hospital for suspected and mild cases of COVID-19 infected psychiatric patients in Chengdu, the CMHC has also established a cooperative plan with designated comprehensive hospitals for joint treatment to achieve seamless connectivity and optimal and quickest control of both COVID-19 and psychiatric symptoms. On the other hand, as the only leading unit of the regional mental health union in Chengdu, the CMHC provides member units with technical support for infection control deployment and organizes them to give onsite and online psychological intervention services in their communities. Given the CMHC's role in the current epidemic, infection control measures addressed in the present study have been implemented and popularized in the member units of the regional mental health union in Chengdu.

Given timeliness, however, only effects of the measures in CMHC were evaluated in the present study. Future work will include intervention data collected in member units of the regional mental health union to further verify the effectiveness of the measures and develop the evidence-based system for infection prevention and control.

Overall, the current research provides a novel insight into the combat against COVID-19 and new evidence for effective hospital infection control during a major epidemic. Some characterized measures, such as the establishment of the transitional isolation ward, air fumigation using traditional Chinese medicine, and psychological guidance and intervention for patients and staff, are worthy of further discussion and diffusion.

## Data Availability Statement

The raw data supporting the conclusions of this article will be made available by the authors, without undue reservation.

## Ethics Statement

The studies involving human participants were reviewed and approved by the Ethics Committee of the Fourth People's Hospital of Chengdu. Written informed consent for participation was not required for this study in accordance with the national legislation and the institutional requirements.

## Author Contributions

MY, MH, and SG contributed to literature search and conception of the study. HW contributed to acquisition of the data. ZL, QZ, and XL analyzed data. MY, HW, MH, and SG interpreted results. MY, MH, and SG wrote the paper. All authors discussed the results and commented on the manuscript.

## Conflict of Interest

The authors declare that the research was conducted in the absence of any commercial or financial relationships that could be construed as a potential conflict of interest.
